# Prognostic significance of solitary lymph node metastasis in patients with squamous cell carcinoma of middle thoracic esophagus

**DOI:** 10.1186/1477-7819-10-210

**Published:** 2012-10-04

**Authors:** Jie Wu, Qi-Xun Chen, Xing-Ming Zhou, Wei-Ming Mao, Mark J Krasna, Li-Song Teng

**Affiliations:** 1 Department of Surgical Oncology, First Affiliated Hospital, Zhejiang University School of Medicine, 79 Qingchun Road, Hangzhou 310033, China; 2 Department of Thoracic Surgery, Zhejiang Cancer Hospital, Hangzhou, 310022, China; 3 Meridian Cancer Care, Jersey Shore University Medical Center, Neptune, NJ, 07753, USA

**Keywords:** Esophageal cancer, Solitary lymph node, Prognosis, Lymphadenectomy

## Abstract

**Background:**

The aim of this study is to compare clinical outcomes between patients with solitary lymph node metastasis and node-negative (N0) patients in squamous cell carcinoma of the middle thoracic esophagus.

**Methods:**

A series of 135 patients with squamous cell carcinoma of the middle thoracic esophagus were retrospectively investigated. There were 33 patients with solitary lymph node metastasis and 102 N0 patients. Skip metastasis in 33 patients with solitary lymph node metastasis was defined according to three criteria: Japanese Society for Esophageal Disease (JSED), American Joint Commission on Cancer (AJCC), and the anatomical compartment.

**Results:**

In 33 patients with solitary lymph node metastasis, skip metastasis was shown in 13, 23, and 8 patients according JSED, AJCC and anatomical compartment respectively. The 5-year survival rates for N0 patients and patients with solitary lymph node metastasis were 58% and 32% respectively (***P*** =0.008). Multivariate analysis revealed that skip metastasis was not an independent prognostic factor.

**Conclusions:**

For patients with middle thoracic esophageal squamous cell carcinoma, solitary lymph node metastasis has a negative impact on survival compared with N0 disease; skip metastasis, however, is comparable to N0 diseases in predicting prognosis.

## Background

Middle thoracic esophageal squamous cell carcinoma is characterized by a bidirectional lymphatic spread and lymph node metastasis [1,2]. Lymphadenectomy is an important component of surgical procedures aiming for complete resection of lesions for patients with esophageal carcinoma. The sentinel lymph node concept for lymphadenectomy has recently been developed in order to individualize the indication for lymphadenectomy and therefore to reduce the surgical stress of thoracic esophageal carcinoma surgery [3]. However, this concept is questioned by some authors [4-6]. For example, the site of solitary lymph node metastasis, which is thought to be the site of initial lymph node metastasis, is difficult to predict in esophageal carcinoma because the location of solitary lymph node metastasis is extensively distributed in the neck, mediastinum, and upper abdomen [4-6]. Similarly, metastasis to anatomically distant lymph nodes, known as skip metastasis, could develop even in the early phase of lymphatic invasion in patients with esophageal carcinoma [4,5,7,8].

The number of metastatic lymph nodes is one of the most important prognostic factors in esophageal cancer [9]. The new seventh tumor, node, metastasis (TNM) staging system reclassified the N stage according to the number of metastatic lymph nodes [10]. Esophageal carcinoma has been regarded as in the earliest lymph node invasion when only one lymph node is involved [4,8]. It is important to investigate the prognosis of solitary lymph node metastasis before the presence of multiple metastatic nodes, which has already meant systematic disease and poor prognosis [11]. However, there are few reports analyzing the prognosis of solitary lymph node metastasis in esophageal cancer.

In this study, the clinical outcomes of 33 patients with solitary lymph node metastasis in squamous cell carcinoma of the middle thoracic esophagus were retrospectively investigated and compared with node-negative (N0) patients to evaluate the prognostic significance.

### Patients and methods

#### Patients

Between January 2003 and December 2009, a series of 235 patients with middle thoracic esophageal squamous cell carcinoma underwent curative esophagectomy in the Department of Thoracic Surgery, Zhejiang Cancer Hospital, Hangzhou, China. Patients who had a history of cancer, had a synchronous cancer, or had previously received chemotherapy or radiotherapy were not included in this series. Of the 235 patients, 33 had solitary lymph node metastasis, and 102 patients had no lymph node metastasis. These 135 patients were analyzed in this retrospective study. The institutional review board of Zhejiiang Cancer Hospital and First Affiliated Hospital, Medical College, Zhejiang University approved the study and the need for individual patient consent was waived.

### Preoperative evaluation

All patients had endoscopy with biopsy, barium swallow examination, computed tomography of the chest and upper abdomen, and ultrasound of the neck. Pathological diagnosis of squamous cell carcinoma was confirmed before the operation. Pulmonary and cardiac function testing were routinely performed to assess medical operability. Tumor location was defined according to the 6th edition of the American Joint Commission on Cancer (AJCC) Cancer Staging Manual [12]. Recurrent laryngeal nerve palsy and the presence of clinical supraclavicular or cervical nodal involvement were considered a contraindication for surgery. The patients underwent surgery after completing the preoperative evaluation and providing written informed consent.

### Surgical procedure

In our institute, two types of lymphadenectomy were carried out as a standard procedure for middle thoracic esophageal cancer. The choice of lymphadenectomy depended on the surgeon’s preference. Four surgeons performed two-field lymphadenectomy while two performed three-field lymphadenecotmy. The surgical indication was the same for operations using these two types of lymphadenectomy.

All patients underwent right transthoracic esophagectomy with either two- or three-field lymphadenectomy. In principle, two-field lymphadenectomy included total mediastinal and upper abdominal lymphadenectomy. Three-field lymphadenectomy added resection of cervical paratracheal, cervical paraesphageal and supraclavicular lymph nodes to two-field lymphadenectomy. Gastrointestinal reconstruction was achieved by stomach bypass through the posterior mediastinal route and anastomosis was performed in the neck for all patients. After surgery, all removed nodes were labeled by their anatomical locations by the operating surgeon.

### Definition of skip metastasis

Three criteria were taken to define skip metastasis as follows: 1. Lymph node classification based on the guidelines of the Japanese Society for Esophageal Disease (JSED), which divides the locations of lymph node metastases into four categories (N1 through N4) [13]. Skip metastasis was diagnosed when solitary lymph node metastasis was observed in N2, N3 or N4 beyond N1. For a middle thoracic tumor, skip metastasis was defined when metastatic lymph node occurred in any locations other than 106rec and 108 (Table 1). 2. Lymph node classification based on the 6th edition of the AJCC Cancer Staging Manual [12], which numbers the location of lymph node metastasis. For a middle thoracic tumor, skip metastasis was defined when solitary lymph node metastasis was observed in the upper mediastinum, upper abdomen and neck, that is, metastatic lymph node that occurred in any locations other than station 5 through 10 (Table 2). 3. Lymph node classification based on the anatomical compartment. Skip metastasis was diagnosed when a solitary lymph node metastasis was observed in the abdomen and neck rather than in the mediastinum.

**Table 1:** Lymph node classification of middle thoracic carcinoma based on the guidelines of the Japanese Society for Esophageal Disease (JSED)

**Category**	**Numbering**	**Station**
N1	108	Middle thoracic paraesophageal
	106rec	Recurrent nerve
N2	101	Cervical paraesophageal
	105	Upper thoracic paraesophageal
	106tbL	Left tracheobronchial
	107	Subcarinal
	109	Main bronchus
	110	Lower paraesophageal
	1	Right cardiac
	2	Left cardiac
	3	Lesser curvature
	7	Left gastric artery
N3	104	Supraclavicualr
	111	Supradiaphragmatic
	112	Posterior mediastinal
	20	Esophageal hiatus of the diaphragm
N4	100	Superficial cervical
	102	Deep cervical
	103	Peripharyngeal
	106pre	Pretracheal
	106tbR	Right tracheobronchial
	113	Bottalo
	114	Anterior mediastinal
	4	Greater curvature
	5	Suprapyloric
	Others	

**Table 2:** Lymph node classification of middle thoracic carcinoma based on AJCC

**Region**	**Numbering**	**Station**
Middle and lower mediastinum	7	Subcarinal
	8 M	Middle paraesophageal
	8 L	Lower paraesophageal
	9	Pulmonary ligament
	10R	Right tracheobronchial
	10 L	Left tracheobronchial
	15	Diaphragmatic
Upper mediastinum	2R	Right upper paratracheal
	2 L	Left upper paratracheal
	3P	Posterior mediastinal
	4R	Right lower paratracheal
	4 L	Left lower paratracheal
	5	Aortopulmonary
	6	Anterior mediastinal
Neck	1	Supraclavicular
Upper abdomen	16	Paracardial
	17	Left gastric
	18	Common hepatic
	19	Splenic
	20	Celiac

### Follow-up

In general, a follow-up examination was performed in our outpatient department every 3 months for the first 2 years and 6 months thereafter. The routine follow-up examination included a physical and routine blood examinations, blood chemistry, measurement of tumor markers (carcinoembryonic antigen, squamous cell carcinoma antigen), radiograph of the chest, and ultrasound. Computed tomography of the chest and upper abdomen were done every 6 months. Endoscopy was done yearly. Complete follow-up information until death or March 2012 was available for all patients.

### Statistical analysis

The distribution of continuous variables was described with the median and range. Categorical variables were compared between groups using the chi-square test and Fisher’s exact test if necessary. Survival curves were constructed by the Kaplan-Meier method [14] and the log-rank test [15] was used to determine significance. Significant variables identified by univariate analyses were assessed by multivariate analyses using Cox’s proportional hazard model [16]. A backward method was used to eliminate insignificant variables. ***P*-** values less than 0.05 were considered statistically significant.

## Results

### Comparison of clinicopathological characteristics between patients with solitary lymph node metastasis and patients with no lymph node metastasis (N0)

Clinicopathological characteristics of these 135 patients with N0 or solitary lymph node metastasis are listed in Table 3. In patients with solitary lymph node metastasis, advanced tumors (T3) were significantly more common than in N0 patients. There was no significant difference in any other variables between these two groups. However, if the same comparison was made among the entire 235 patients, who were divided into three groups including N0 patients, patients with solitary lymph node metastasis and patients with more than one lymph node metastasis, all variables except age and sex were significantly different among the three groups. Tumor length > 5 cm, G3, T3 and T4 stage and intramural metastasis were more frequent in patients with more than one lymph node metastasis than in the other two groups of patients.

**Table 3:** Comparison of clinicopathological characteristics between patients with solitary lymph node metastasis and N0 patients

**Characteristic**	**Solitary lymph node metastasis (n = 33)**	**N0 (n = 102)**	***P*-value**
Age (years)			0.253
≤ 60	24	63	
> 60	9	39	
Sex			0.743
Female	7	19	
Male	26	83	
Tumor length			0.839
≤ 5 cm	24	76	
> 5 cm	9	26	
Differentiation			0.007
G1	4	34	
G2	21	60	
G3	8	8	
T category			< 0.001
T1	2	28	
T2	2	24	
T3	29	50	
Intramural metastasis			1.000
No	32	99	
Yes	1	3	
			0.739
≤ 25	18	59	
> 25	15	43	
Adjuvant therapy			0.068
No	26	93	
Yes	7	9	
Lymphadenectomy			0.276
2-field	29	81	
3-field	4	21	

### Distribution of solitary lymph node metastasis

The distribution of solitary lymph node metastasis based on JSED, AJCC and anatomical compartment is summarized in Table 4 and Table 5. Recurrent nerve node (12 cases) and middle thoracic paraesophageal node (eight cases) were the most common metastatic sites in solitary lymph node metastasis. Skip metastasis was shown in thirteen, twenty-three, and eight patients according JSED, AJCC and anatomical compartment respectively.

**Table 4:** **Distribution of solitary lymph node metastasis in 33 patients based on the guidelines of the Japanese Society for Esophageal Disease (JSED) compared with the American Joint Commission on Cancer** (**AJCC)**

	**AJCC**									
	**Skip (−) (n = 10)**			**Skip (+) (n = 23)**						
	**7**	**8 M**	**8 L**	**2R**	**2 L**	**3P**	**4**	**16**	**17**	**1**
JSED	-	-	-	-	-	-	-	-	-	-
Skip (−) (n = 20)	-	-	-	-	-	-	-	-	-	-
106recR	-	-	-	10	-	-	-	-	-	-
106recL	-	-	-	-	2	-	-	-	-	-
108	-	8	-	-	-	-	-	-	-	-
Skip (+) (n = 13)										-
105	-	-	-	-	-	2	-	-	-	-
107	1	-	-	-	-	-	-	-	-	-
110	-	-	1	-	-	-	-	-	-	-
1	-	-	-	-	-	-	-	2	-	-
2	-	-	-	-	-	-	-	1	-	-
3	-	-	-	-	-	-	-	-	1	-
7	-	-	-	-	-	-	-	-	2	-
104R	-	-	-	-	-	-	-	-	-	1
104 L	-	-	-	-	-	-	-	-	-	1
106pre	-	-	-	-	-	-	1	-	-	-

**Table 5:** Summary of solitary lymph node metastasis in thirty-three patients defined by three criteria

	**JSED**		**AJCC**		**Anatomy**	
	**Skip (−) (n = 20)**	**Skip (+) (n = 13)**	**Skip (−) (n = 10)**	**Skip (+) (n = 23)**	**Skip (−) (n = 25)**	**Skip (+) (n = 8)**
upper mediastinum	12	3		15	15	
Middle mediastinum	8	1	9		9	
Lower mediastinum		1	1		1	
Upper abdomen		6		6		6
Cervix		2		2		2

JSED, Japanese Society for Esophageal Disease; AJCC, American Joint Commission on Cancer.

### Survival

**Figure 1:**
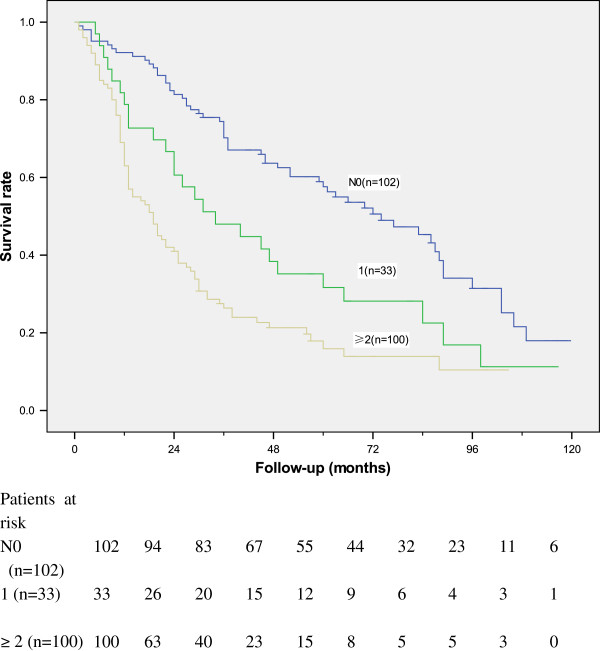
**Survival curves in 235 patients according to lymph node status (*P *< 0.01).** N0 vs. one positive node, *P* = 0.008; N0 vs. two or more positive nodes, *P* < 0.001; one positive node vs. two or more positive nodes, *P* = 0.048.

The 5-year survival rates for N0 patients, patients with solitary lymph node metastasis and patients with ≥ two lymph node metastases were 58%, 32%, and 15.9% respectively (***P*** < 0.001) (Figure 1). There were significant differences in survival between N0 patients and patients with solitary lymph node metastasis (***P*** = 0.008), between patients with solitary lymph node and with ≥ two lymph node metastases (***P*** = 0.048), and between N0 patients and patients with ≥ two lymph node metastasis (***P*** < 0.001).

**Figure 2:**
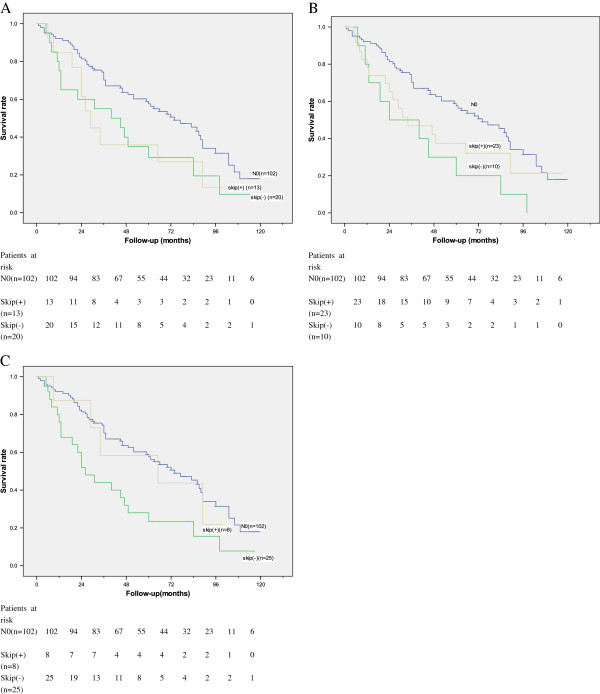
**Survival curves in patients with no metastases (N0 patients), patients with skip metastasis, and patients without skip metastasis according to different criteria.** (**A**) JSED (N0 vs. skip (+), *P* = 0.047; N0 vs. skip (−), *P* = 0.031; skip (+) vs. skip (−), *P* = 0.302). (**B**) Survival curves in N0 patients, patients with skip metastasis, and patients without skip metastasis according to AJCC (N0 vs. skip (+), *P* = 0.094); N0 vs. skip (−), *P* = 0.004; skip (+) vs. skip (−), *P* = 0.302). (**C**) Survival curves in N0 patients, patients with skip metastasis, and patients without skip metastasis according to the anatomical compartment (N0 vs. skip (+), *P* = 0.74; N0 vs. skip (−), *P* = 0.002; skip (+) vs. skip (−), *P* = 0.180).

Patients with solitary lymph node metastasis were further analyzed. According to the JSED, the 5-year survival rates for skip (+) and skip (−) patients were 36% and 29% respectively, both of which were significantly different from the 5-year survival for N0 patients (***P*** = 0.047 and ***P*** = 0.031 respectively) (Figure 2A). According to the AJCC, the 5-year survival rates for skip (+) and skip (−) patients were 37% and 20% respectively. There was a significant difference in survival between skip (−) patients and N0 patients (***P*** = 0.004); however, there was no significant difference in survival between skip (+) patients and N0 patients (***P*** = 0.094) (Figure 2B). According to the anatomical compartment, the 5-year survival rates for skip (+) and skip (−) patients were 58% and 23% respectively. Survival of Skip (+) patients was not significantly different from that of N0 patients (***P*** = 0.740). There was a significant difference in survival between N0 patients and skip (−) patients (***P*** = 0.002) (Figure 2C).

### Univariate and multivariate survival analysis

Univariate survival analysis showed that T category and N status were significantly associated with prognosis (Table 6). Multivariate analysis was performed including T category and N status. Owing to the different criterion of skip metastasis, three models were constructed. In a model according to the JSED, T category was the independent prognostic factor. In the other two models according to the AJCC and anatomical compartment, N status was the independent prognostic factor. In these two models, however, skip metastasis was not significantly different from N0 in predicting prognosis (Table 7).

**Table 6:** Univariate analysis of survival in 135 patients with squamous cell carcinoma of the middle thoracic esophagus

**Variable**	**Number of patients**	**5-year survival rate**	**Median survival (months)**	***P*-value**
Age (years)				0.939
≤ 60	87	51%	63	
> 60	48	52%	61	
Sex				0.500
Female	26	48%	63	
Male	169	52%	60	
Tumor length				0.903
≤ 5 cm	100	51%	63	
> 5 cm	35	50%	88	
Differentiation				0.553
G1	38	59%	74	
G2	81	48%	59	
G3	16	49%	60	
T category				0.041
T1	30	67%	86	
T2	26	55%	77	
T3	79	44%	45	
Intramural metastasis				0.135
No	131	52%	63	
Yes	4	25%	29	
Number of dissected lymph nodes				
≤ 25	77	53%	66	0.929
> 25	58	48%	60	
Lymphadenectomy				0.206
2-field	110	48%	60	
3-field	25	66%	87	
N				
Negative	102	58%	74	0.008
Positive	33	32%	34	

**Table 7:** Multivariate analysis of survival in 135 patients with squamous cell carcinoma of the middle thoracic esophagus

**Variable**	**Hazard ratio**	**95% CI**	***P*-value**
JESD			
T category			0.047
T1 (reference)	1		
T2	1.442	0.685, 3.033	0.335
T3	2.060	1.126, 3.769	0.019
N			0.185
N0 (reference)	1		
Skip (+)	1.586	0.780, 3.223	0.203
Skip (−)	1.610	0.900, 2.882	0.109
AJCC			
T category			0.205
T1 (reference)	1		
T2	1.474	0.700, 3.105	0.308
T3	1.768	0.943, 3.317	0.076
N			0.013
N0 (reference)	1		
Skip (+)	1.591	0.915, 2.769	0.100
Skip (−)	2.533	1.291, 4.970	0.007
Anatomy			
T category			0.156
T1 (reference)	1		
T2	1.517	0.720, 3.918	0.273
T3	1.843	0.988, 3.433	0.055
N			0.010
N0 (reference)	1		
Skip (+)	1.145	0.458, 2.860	0.772
Skip (−)	2.178	1.320, 3.593	0.002

JSED, Japanese Society for Esophageal Disease; AJCC, American Joint Commission on Cancer; CI, confidence interval.

## Discussion

Patients with solitary lymph node metastasis in esophageal cancer has been considered a distinct prognostic subgroup with cancer outcomes closer to node-negative disease than any other node-positive subgroup [17]. It has even been believed that there is no difference in survival between patients with solitary lymph node metastasis and N0 patients with esophageal squamous cell carcinoma, and that solitary lymph node metastasis does not affect the prognosis [5]. In this report, except for T category, all clinicopathological variables did not differ between N0 patients and patients with solitary lymph node metastasis. Solitary lymph node metastasis had a worse survival compared with N0 disease. These results may partly explained by the fact that a majority of patients with solitary lymph node metastasis were at the T3-stage. But skip metastasis was not a risk factor for poor prognosis. According to the JSED criterion both skip (+) and skip (−) patients showed worse survival than N0 patients, but it was T category not N status that was the independent prognostic factor in multivariate analysis. Although N status was the independent prognostic factor according to the other two criteria, there was still no significant difference between N0 and skip metastasis in predicting prognosis.

The prognostic impact of lymph node skip metastasis in esophageal carcinoma has been unclear. Some reports have shown a favorable prognosis of skip metastasis (compared with adjacent or continuous lymph node metastasis) and others have not [7,18]. Prenzel et al. [7] attributed the different results to the different lymph node classification used to define skip metastasis. In this series, three criteria were used to define skip metastasis. Skip metastasis did not show a favorable prognosis compared with adjacent metastasis. But multivariate analysis did show that skip metastasis was not a prognostic factor, whereas adjacent metastasis was prognostic in two of the three models. It must be noted that skip metastasis could be caused by inefficient histopathologic examination by missing out small metastasis in positive nodes. In addition, micrometastasis detected by immunohistochemistry in histologically tumor-free nodes is not a rare event in esophageal cancer [6,18]. Natsugoe et al. [6] reported micrometastasis was found in 33 of 59 patients (55.9%) with esophageal carcinoma, who were diagnosed as N0 by routine histological examination.

As previous reports have shown [4-6,8], solitary lymph node metastasis in this report was widely distributed, but was mainly limited to the recurrent nerve region and middle thoracic paraesophagus region. It was reasonable that the JSED classified these two regions as N1 in middle thoracic squamous cell carcinoma. Some authors have found that solitary lymph node metastasis in esophageal carcinoma is predominately located in the abdomen along the lesser curvature and the left gastric artery [2]. But their series included lower thoracic tumors and upper mediastinal lymphadenectomy was not routinely performed. The wide distribution of solitary lymph node metastasis was closely associated with the tumor site and unique features of lymphatic spread from the esophagus, which has been discussed in many previous reports [4,5,8,19,20]. In addition, middle thoracic tumors have a more obvious tendency to develop bidirectional metastasis than upper and lower thoracic tumors of the esophagus [1,19,20], so it is still hard to predict the location of solitary lymph node metastasis, even if sentinel navigation surgery is used [4].

Three-field might be more appropriate than two-field lymphadenectomy for middle thoracic esophageal squamous cell carcinoma in terms of the wide distribution of solitary lymph node metastasis. But there was no survival difference between patients treated with these two types of lymphadenectomies (data not shown). Many similar results reported earlier have also been summarized in reviews of the literature [21,22]. So far, controversy exists as to the extent of lymphadenectomy required in esophageal cancer. There is no doubt that more extensive lymphadenectomy provides more accurate nodal staging [21], but whether it improves survival still needs to be tested in well-designed randomized controlled trials [22].

Several potential shortcomings in this retrospective study deserve attention. Surgeons chose different types of lymphadenectomy depending on their preferences. A certain selection bias can therefore not be excluded. It is also possible that variation in the quality of lymphadenectomy among different surgeons may interfere with the results. In addition, the sample size was limited by nature of the single institution of the study. Validation from other institutions is needed. Nonetheless, this series proved to be homogenous with regard to many clinical variables, such as tumor site, pathological type, and preoperative management.

## Conclusions

For patients with middle thoracic esophageal squamous cell carcinoma, solitary lymph node metastasis has a negative impact on survival compared with N0 disease; skip metastasis, however, is comparable to N0 disease in predicting prognosis.

## Competing interests

The authors declare that they have no competing interests.

## Authors’ contributions

JW conceived this study, collected data, performed analysis and drafted the manuscript. QXC participated in study design, literature search and coordination. JW, QXC, XMZ and WMM participated in the treatment of these patients. MJK performed data analysis and helped to draft the manuscript. LST participated in study design and helped to draft the manuscript. All authors read and approve the final manuscript.
